# Characterization of the Secreted Acid Phosphatase SapS Reveals a Novel Virulence Factor of *Staphylococcus aureus* That Contributes to Survival and Virulence in Mice

**DOI:** 10.3390/ijms232214031

**Published:** 2022-11-14

**Authors:** Nour Ahmad-Mansour, Mohamed Ibrahem Elhawy, Sylvaine Huc-Brandt, Nadhuma Youssouf, Linda Pätzold, Marianne Martin, Noran Abdel-Wadood, Ahmad Aljohmani, Madjid Morsli, Gabriela Krasteva-Christ, Sören L. Becker, Daniela Yildiz, Jean-Philippe Lavigne, Laila Gannoun-Zaki, Markus Bischoff, Virginie Molle

**Affiliations:** 1 Laboratory of Pathogen Host Interactions, University of Montpellier, Centre National de la Recherche Scientifique, UMR 5235, 34095 Montpellier, France; 2 Institute for Medical Microbiology and Hygiene, University of Saarland, 66421 Homburg, Germany; 3 Institute of Anatomy and Cell Biology, Saarland University, 66421 Homburg, Germany; 4 Institute of Experimental and Clinical Pharmacology and Toxicology, Saarland University, 66421 Homburg, Germany; 5 Virulence Bactérienne et Infections Chroniques, Department of Microbiology and Hospital Hygiene, CHU Nîmes, University of Montpellier, INSERM U1047, 30029 Nîmes, France

**Keywords:** *Staphylococcus aureus*, acid phosphatase, SapS, macrophage survival, virulence, inflammatory response, biofilm, oxidative stress

## Abstract

*Staphylococcus aureus* possesses a large arsenal of immune-modulating factors, enabling it to bypass the immune system’s response. Here, we demonstrate that the acid phosphatase SapS is secreted during macrophage infection and promotes its intracellular survival in this type of immune cell. In animal models, the SA564 *sapS* mutant demonstrated a significantly lower bacterial burden in liver and renal tissues of mice at four days post infection in comparison to the wild type, along with lower pathogenicity in a zebrafish infection model. The SA564 *sapS* mutant elicits a lower inflammatory response in mice than the wild-type strain, while *S. aureus* cells harbouring a functional *sapS* induce a chemokine response that favours the recruitment of neutrophils to the infection site. Our *in vitro* and quantitative transcript analysis show that SapS has an effect on *S. aureus* capacity to adapt to oxidative stress during growth. SapS is also involved in *S. aureus* biofilm formation. Thus, this study shows for the first time that SapS plays a significant role during infection, most likely through inhibiting a variety of the host’s defence mechanisms.

## 1. Introduction

*Staphylococcus aureus* is an important human pathogen that has been linked to high rates of mortality and morbidity across a diverse range of illnesses that may be acquired in hospitals or in the community [1,2]. *S. aureus* infection can manifest itself clinically in a variety of ways, ranging from mild skin infections to a potentially fatal form of bacteremia that can lead to complications including endocarditis and pneumonia. Because it has such many different virulence factors, *S. aureus* is capable of adhering to and invading a wide variety of tissue which contributes to its sophisticated human adaption [3,4,5]. *S. aureus* is also capable of producing a large range of secreted proteins that participate in the pathogen host-interaction mechanisms and especially with the immune system, corresponding to the first line of defence against invading bacteria [6]. It is interesting to note that the presence of this diversity of virulence factors allows *S. aureus* to colonize a variety of environments and establish infections by distinct pathways. Throughout these infections, *S. aureus* and the host cells engage in a competition for nutrients and reorganize their metabolism in an effort to survive. This metabolic interaction and crosstalk are what ultimately decides the fate of the infection by modifying metabolic processes in host immune cells. The activation of antimicrobial responses may lead ultimately to the elimination of bacteria and can include cell death processes. This highlights the relevance of metabolic alterations in both host and bacterial cells in determining the course of staphylococcal infections as recently reviewed in [7,8,9]. Although a lot of knowledge has been gathered from research aiming at identifying the pathophysiological processes that occur during an infection caused by *S. aureus* in macrophages, the bacterial virulence factors and signalling mechanisms that lead to these changes are still only partially known [10].

In recent years, we and others have shown that some host-pathogen interactions involve phosphatase enzymes that are released by pathogens [11,12,13,14,15,16]. We recently showed that PtpA, a *S. aureus*-produced low-molecular-weight protein tyrosine phosphatase [17], was secreted during growth and was involved in the pathogen’s virulence [16]. Interestingly, the new class C non-specific acid phosphatase SapS (also known as SAcP), corresponding to another family of protein phosphatase than PtpA, was identified as secreted by *S. aureus* strain 154 isolated from vegetables [18]. Acid phosphatases are found almost everywhere in nature and are responsible for the hydrolysis of the phosphoryl groups of phosphomonoesters at an acidic pH [19]. Both prokaryotes and eukaryotes need these enzymes for the generation, assimilation, and the mobilisation of inorganic phosphate, in addition to being important for the phosphorelay systems that play a role in the signal transduction pathways. Acid phosphatases from other pathogens were reported to be involved in reduction of the respiratory burst, which suggests that these acid phosphatases have a role in the virulence of these pathogens [13,20,21,22,23,24,25,26]. However, while SapS was characterised biochemically [18], its impact on immune escape, infectiousness and virulence has not yet been evaluated.

Here, we report that the deletion of *sapS* in the *S. aureus* strain SA564 significantly reduced the mutant’s ability to escape intracellular killing by RAW 264.7 macrophages. Additionally, we show that SapS can be found outside of bacterial cells ingested by macrophages. In animal models, the SA564 *sapS* mutant produced a significantly lower bacterial burden in liver and renal tissues of mice at four days post infection in comparison to the wild type, as well as a lower virulence in the zebrafish model of infection. SA564 *sapS* cells also displayed an enhanced susceptibility to hydrogen peroxide challenge, although only little impact of the *sapS* mutation regarding the transcription of genes that are implicated in oxidative stress adaption process was observed. An aspartic acid to alanine exchange in the active site of SapS revealed furthermore that SapS phosphatase function is neither necessary for *S. aureus* to survive in macrophages nor for infectivity in the zebrafish infection model.

## 2. Results

### 2.1. SapS Is Involved in Intramacrophage Survival of S. aureus

Considering how crucial the previously identified phosphatases PtpA and PtpB are to the capacity of *S. aureus* to survive inside macrophages [16,27], we hypothesised that SapS, which is highly conserved in *S. aureus* (Figure S1), might have a similar impact. An in silico investigation yielded that until 5 November 2022, the *sapS* encoding gene of *S. aureus* isolate SA564 exhibited 100% coverage and 100% identity with all *S. aureus* genomes sequences by blast against NCBI GenBank database, represented by the 100 hit-blast strains deposited previously on NCBI GenBank database. Phylogenetic analysis of the identified *sapS* complete sequence with the most hit-blasts including the *S. aureus* USA300 strain and other *Staphylococcus* species obtained by blast after exclusion of the *S. aureus* TaxID, confirmed that the *sapS* gene remains well conserved in all *S. aureus* isolates at 100% sequence similarity (Figure S1).

**Figure 1:**
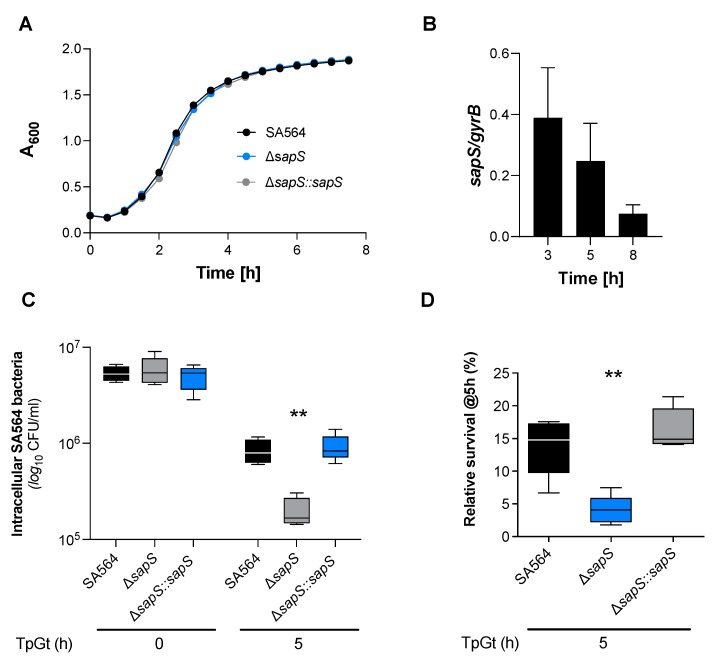
SapS has no impact on *S. aureus* SA564 growth *in vitro* but contributes to intramacrophage survival. (**A**) Growth kinetics of *S. aureus* strains SA564 (black symbols), SA564 Δ*sapS* (blue symbols), and SA564 Δ*sapS::sapS* (grey symbols) in TSB. Cells were cultured at 37 °C and 150 rpm in 96-well plates using a Spark 20M (Tecan, Trading AG, Männedorf, Switzerland) microplate reader. Data represent the mean A_600_ readings ± SD at the time points indicated (n = 3; error bars are too small to be visible). (**B**) Transcription of *sapS* during growth in TSB. Cells of SA564 were cultured at 37 °C and 225 rpm in a culture to flask volume of 1:10, and after 3 h, 5 h, and 8 h of growth, cells were harvested, total RNAs isolated, and qRT-PCRs performed for *sapS*. Transcripts were quantified in reference to gyrase B mRNA. Data are presented as mean + SD of five biological replicates. (**C**,**D**) Survival of *S. aureus* strain derivatives in infected RAW 264.7 macrophages. CFU rates recovered from infected cells lysates at the time points indicated. Data are presented as mean + SD (n = 5 biological replicates) (**C**) and survival rates expressed in relationship to the intracellular bacterial cell counts that were detected immediately after the gentamicin/lysostaphin treatment (T0), which was set to 100%. The data are displayed as box and whisker plots, displaying the median (horizontal line), interquartile range (25–75% box), and standard deviation (bars) of five different experiments (**D**). **, *p* < 0.01 (Kruskal–Wallis test followed by Dunn’s post-hoc test). TpGt, time post gentamicin treatment.

To investigate this hypothesis, a *sapS* deletion mutant (SA564 Δ*sapS*) and its cis-complementation derivative SA564 Δ*sapS::sapS* were constructed in SA564, a clinical isolate of clonal complex 5 (CC5). We first conducted *in vitro* growth curves using the wild type, mutant, and complemented strains to see if the loss of *sapS* in *S. aureus* would have an impact on bacterial growth in suspension. There was no noticeable impact on the growth in tryptic soy broth (TSB) caused by the deletion of *sapS* in *S. aureus* strain SA564, as shown in Figure 1A. To get an idea about the expression of SapS in *S. aureus* SA564, we next evaluated the transcription of *sapS* while the bacteria were grown in TSB (Figure 1B). The relative abundance of *sapS* transcripts clearly decreased over time, suggesting that SapS is predominantly expressed in SA564 during the early growth stages.

**Figure 2:**
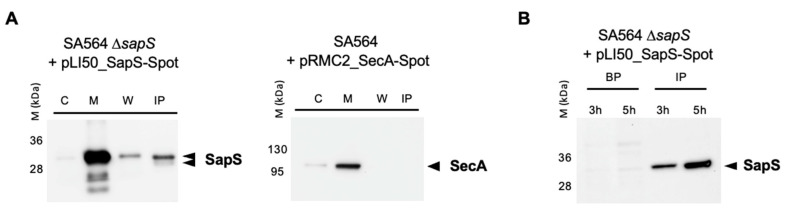
SapS is produced by *S. aureus* both *in vitro* and in infected macrophages. (**A**) Cultures were grown up to exponential phase and supernatants from SA564 ∆*sapS* + pLI50_SapS-Spot and SA564 + pLI50_SecA-Spot (negative secretion control) were filtered and immunoprecipitated using anti-Spot magnetic beads, while bacterial pellets were resuspended and lysed in PBS with lysostaphin, a protease inhibitor cocktail, DNAase I and fractionated. Cytoplasm (C), membrane (M), cell wall (W), and immunoprecipitated proteins (IP) from supernatants were migrated on SDS-PAGEs, transferred to PVDF membranes for Western-blot analyses using monoclonal mouse anti-Spot antibody as primary antibody (Chromotek, Germany) and an HRP-coupled donkey-anti-mouse antibody as secondary antibody (Jackson Immuno Research). Data are representative of three different experiments. (**B**) RAW 264.7 macrophages (2.5 × 10^5^ cells/well) were infected with *S. aureus* at a MOI of 20, and non-phagocytosed bacteria were subsequently removed by gentamicin/lysostaphin treatment. Infected macrophages were lysed in 0.1% Triton X-100 and centrifuged at 14,000× *g*. The macrophage lysates were immunoprecipitated with anti-Spot magnetic beads (Chromotek), while the pellets of the intracellular bacteria were resuspended in an equal volume of lysis buffer. Immunoprecipitated proteins (IP) and bacterial pellets (BP) were separated on SDS-PAGE and observed with an anti-Spot antibody as described in (**A**). Data are representative of three different experiments (M kDa: molecular markers).

Given that SapS (SACOL0303) includes a 31-aa long signal sequence at the N-terminus of its protein sequence [28] and that the protein was found repeatedly in *S. aureus* cell culture supernatants [18,29,30], one can assume safely that SapS is secreted by *S. aureus* into the extracellular milieu under *in vitro* conditions. However, as it is not known yet whether *S. aureus* also secretes SapS when exposed to an infection-mimicking environment, we created a SA564 Δ*sapS* derivative harbouring plasmid pLI50_SapS-Spot and cultured it in TSB or used it to infect RAW 264.7 macrophages. Next, the presence of SapS-Spot was determined in the supernatants (by immunoprecipitation with anti-Spot magnetic beads; IP) and cytosol-, membrane-, and cell wall-fractions of *S. aureus* cell suspensions grown for 4 h in TSB (Figure 2A), or in lysed macrophage supernatants and intracellular persistent bacteria at 3 and 5 h after infection, respectively (Figure 2B). In *in vitro* cultured *S. aureus* cells, we observed a strong SapS-Spot signal in membrane fractions, and weaker but still clearly visible signals in cell wall fractions and the supernatant, while SapS-Spot was hardly detectable in the cytosol fractions. To rule out that the SapS-Spot signal seen in the supernatants of *S. aureus* TSB cultures was due to a partial lysis of the bacterial cell population, we transformed SA564 Δ*sapS* with plasmid pRMC2_SecA-Spot, which expresses a SecA-Spot derivative known to associate with the membrane fraction of *S. aureus* [27]. In line with its predicted localization, we observed SecA-Spot signals almost exclusively in the membrane fraction, and to a small extent in the cytosol fractions, but neither in the cell wall fractions nor in the supernatants (Figure 2A). These findings suggest that SapS associates predominantly with the bacterial cell membrane and is in part secreted into the extracellular milieu after cleavage of its peptide signal as indicated by a lower SapS migrating band (Figure 2A). Notably, when SapS-Spot expressing SA564 Δ*sapS* cells were used to infect RAW 264.7 macrophages, the majority of SapS-Spot was detectable in macrophage lysates at 3 and 5 h post infection (Figure 2B), suggesting that SapS is primarily secreted into the phagolysosome and/or cytosol of the *S. aureus* loaded immune cells. However, we cannot exclude here that the majority of SapS is released due to bacterial cell lysis under these infection-mimicking culture conditions.

Next, we determined how cells of the *S. aureus* strains SA564, SA564 Δ*sapS*, and SA564 Δ*sapS::sapS* survived inside RAW 264.7 macrophages. Intracellular CFU counts at T0 were similar for all the three bacterial strains in RAW 264.7 cells, indicating that the *sapS* mutation does not affect adhesion and internalisation of *S. aureus* into this immune cell type, while at 5 h post-Gentamycin treatment (pGt), intracellular bacterial loads decreased significantly for the SA564 Δ*sapS* mutant (Figure 1C). Comparison of the relative survival rates at 5 h pGt identified a significantly lower survival of the *S. aureus sapS* mutant inside of the RAW 264.7 cells, while *cis*-complementation of the *sapS* mutant with a functional *sapS* locus restored intracellular survival rates to values equivalent to those seen with the WT strain (Figure 1D). These findings imply that SapS is important for the intracellular survival capability of *S. aureus* inside macrophages.

### 2.2. SapS Participates to the Virulence of S. aureus in the Zebrafish and Murine Abscess Models

**Figure 3:**
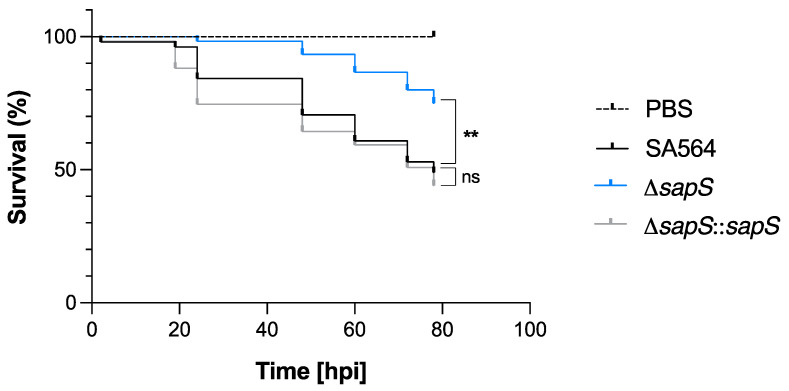
Infected zebrafish embryos display an increased level of survival when infected with the SA564 Δ*sapS* mutant. Kaplan–Meier graphic represents the survival of infected zebrafish embryos after injection in the duct of Cuvier with SA564 (black symbols), SA564 Δ*sapS* (blue symbols), and SA564 Δ*sapS::sapS* (grey symbols) strains at 3 × 10^2^ CFU/nL grown in exponential phase, or PBS (negative control) The proportion of surviving embryos (n = 50 for each, indicative of three different experiments) is represented. **, *p* < 0.01, ns, not significant (Log-rank Mantel–Cox statistical test). hpi, hours post-infection.

Considering that SapS contributes to the survival capability of *S. aureus* inside macrophages (Figure 1C,D), we were curious about the impact of SapS on the bacterium’s ability to cause infection *in vivo*. In order to determine the impact of SapS in *S. aureus* virulence, we used the zebrafish model that has been previously demonstrated to be relevant to monitor *S. aureus* virulence by following the survival rate of infected larvae [31,32]. *S. aureus* SA564 and its isogenic Δ*sapS* derivatives were injected into the duct of Cuvier, respectively, which causes a rapid systemic infection at 50 h post-fertilization, when the zebrafish embryo innate immune system is functional [33]. Infected embryo survival curves revealed that injection of wild-type cells into the larvae killed about 50% of the zebrafish embryos at 80 h post infection (hpi), and a similar killing rate was also observed for larvae infected with the *cis*-complemented Δ*sapS::sapS* derivative (Figure 3). However, when the zebrafish embryos were infected with the SA564 Δ*sapS* mutant, a survival rate of ~80% was observed, suggesting that SapS is an important virulence factor in this model.

**Figure 4:**
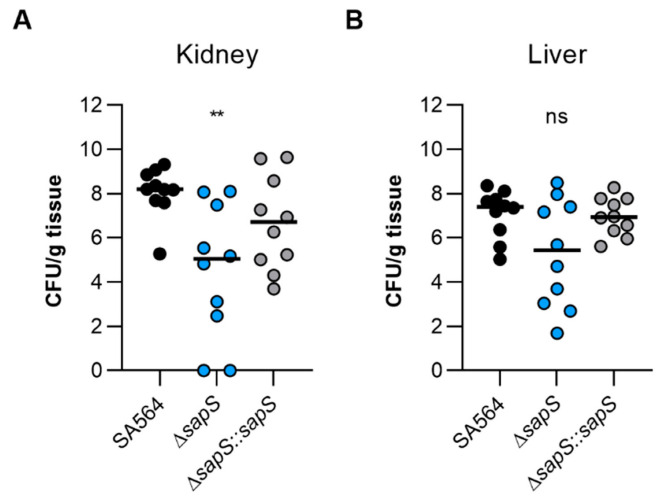
The virulence of *S. aureus* SA564 in a murine abscess model is enhanced by the presence of SapS. C57BL/6N mice were infected via retroorbital injection with 1 x 10^7^ cells of *S. aureus* strain SA564 (black symbols), SA564 Δ*sapS* (blue symbols), and the *cis*-complemented derivative SA564 Δ*sapS::sapS* (grey symbols), respectively (n = 10 per group). Mice were euthanised and to evaluate the bacterial burdens in the kidney (**A**) and liver (**B**) organs, livers and kidneys were excised and homogenised in PBS 4 days after infection. Serial dilutions of the homogenates were then plated on sheep blood agar plates. One mouse is represented by each symbol. The median of all observations is indicated by the horizontal bars. ns, not significant; ** *p* < 0.01 (Kruskal–Wallis test followed by Dunn’s post hoc test). Only differences that are statistically significant between WT and mutants are displayed.

To test the impact of SapS in a mammalian model, we next utilised the *S. aureus*-based liver and kidney abscess model [34]. Once bacteria were injected into the circulating blood of the mice, and bacterial loads in the liver and kidneys were calculated at four days post infection, a significant decrease in viable bacteria was noticed in the kidneys of mice infected with the Δ*sapS* mutant (Figure 4A). Kidney homogenates of SA564 Δ*sapS* challenged mice contained in the median more than 2 *log*_10_ fewer colony forming units (CFU) than kidney homogenates of SA564 infected mice. CFU rates in kidney homogenates of SA564 Δ*sapS::sapS* challenged mice were about 1.5 *log*_10_ higher as those seen with SA564 Δ*sapS* challenged mice, and did not differ statistically from the CFU rates seen in the kidney homogenates of wild-type infected mice. Mice infected with the Δ*sapS* mutant also displayed in the median about 2 *log*_10_ fewer CFU numbers in the liver than wild-type or Δ*sapS::sapS* challenged mice (Figure 4B), despite the fact that this difference was not significantly different according to the statistics (*p* = 0.1873, Kruskal–Wallis test and Dunn’s post-hoc test). Both infection models suggest that SapS is a factor that favorably affects the infectious potential of *S. aureus* under *in vivo* conditions, and that SapS is of particular importance for the bacterium to form abscesses in kidneys.

### 2.3. SapS Modifies the Immune Cell Population in S. aureus-Infected Mice Blood

**Figure 5:**
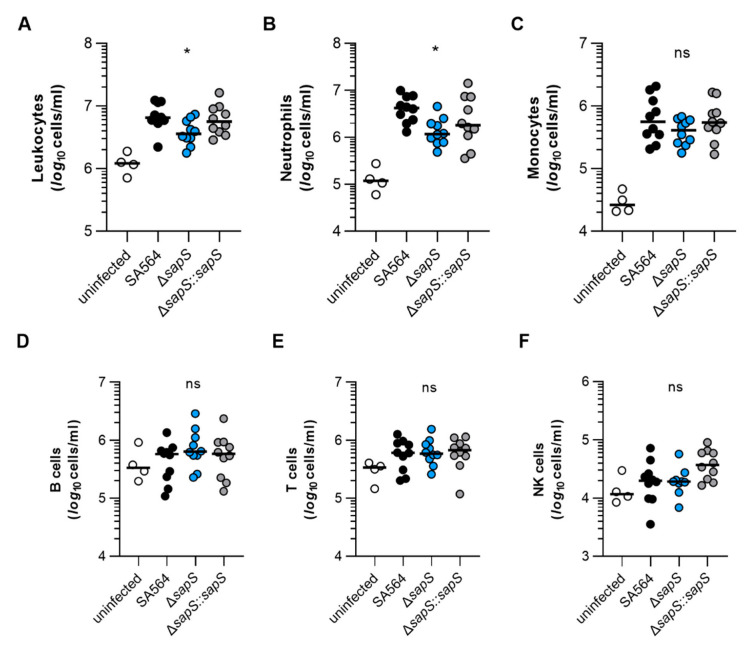
The impact of SapS on the immune cell composition observed in the blood of mice infected with *S. aureus*. C57BL/6N mice were infected with *S. aureus* strain SA564 (black symbols), SA564 Δ*sapS* (blue symbols), and the *cis*-complemented derivative SA564 Δ*sapS::sapS* (grey symbols), respectively (n = 10 per group). After 4 days of infection, blood was withdrawn from narcotized mice, red blood cells lysed, the residual blood cells stained with fluorochrome-conjugated antibodies and the cell contents of leukocytes (**A**), neutrophils (**B**), monocytes (**C**), B cells (**D**), T cells (**E**), and NK cells (**F**) determined by flow cytometry. Blood from age-matched uninfected mice served as control (white symbols). Each symbol represents the cell content obtained from the blood of an individual mouse. The median of all the observations is shown by the horizontal bar. ns, not significant; * *p* < 0.05 (Kruskal–Wallis test followed by Dunn’s post hoc test. Only significant differences between WT-infected and mutant-infected mice are shown).

To get an idea about the impact of the *S. aureus* infection on the murine immune system, we next studied the composition of immune cells in blood of *S. aureus*-infected mice after four days of infection (Figure 5). In comparison to uninfected mice, markedly higher numbers of leukocytes were found in blood of mice infected with the SA564 derivatives (Figure 5A), indicating that all three derivatives allowed for an establishment of an infection. However, when the numbers of leukocytes found in the blood of *S. aureus*-infected mice at four days post infection were compared, a significantly lower level of leukocytes was observed in SA564 Δ*sapS* challenged mice when compared to wild-type infected mice. This change in white blood cell numbers was mainly due to an increase in neutrophils in the blood of wild-type infected mice, which were significantly increased in wild-type infected mice at four days post infection in comparison to SA564 Δ*sapS* challenged mice (Figure 5B), while counts of monocytes, B-cells, T-cells, and NK-cells were not markedly altered between wild-type infected and Δ*sapS* challenged mice (Figure 5C–F). These findings suggest that SA564 elicits a stronger immune response in mice than its isogenic Δ*sapS* derivative.

### 2.4. SapS Affects the KC and MPO Contents in Kidneys of S. aureus-Infected Mice

**Figure 6:**
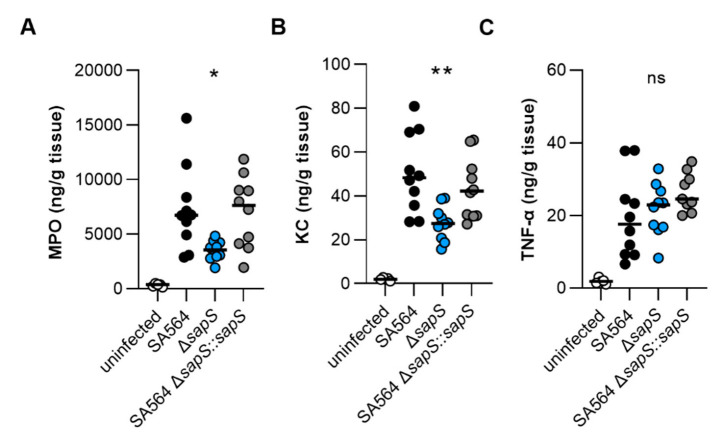
MPO, KC, and TNF-α contents in kidney tissues obtained from mice at day 4 post infection. C57BL/6N mice were infected with *S. aureus* strain SA564 (black symbols), SA564 Δ*sapS* (blue symbols), and the *cis*-complemented derivative SA564 Δ*sapS::sapS* (grey symbols), respectively (n = 10 per group). Four days after infection, mice were euthanised, kidneys were extracted and blended with PBS solution, the tissue homogenates centrifuged, and the MPO (**A**), KC (**B**), and TNF-α (**C**) contents in the supernatants determined by enzyme-linked immunosorbent assays. Kidney homogenates from age-matched uninfected mice served as control (white symbols). Each symbol represents the amount of enzyme/cytokine found in the kidney homogenate of an individual mouse. The median of all the observations is shown by the horizontal bar. ns, not significant; * *p* < 0.05; **, *p* < 0.01 (Kruskal–Wallis test followed by Dunn’s post hoc test. Only significant differences between WT-infected and mutant-infected mice are shown).

To test whether the increased numbers of neutrophils seen in the blood of SA564 infected mice were also accompanied by increased numbers of this immune cell type at the infection side, we next tested the myeloperoxidase (MPO) contents in kidney tissue homogenates obtained from the infected mice at four days post infection. In accordance with the increased numbers of neutrophils in blood of WT and Δ*sapS::sapS* infected mice, we observed in the median twice as high MPO levels as in kidney tissue homogenates obtained from Δ*sapS* infected mice (Figure 6A). Similarly, significantly lower levels of the neutrophil chemoattractant chemokine KC were observed in kidney tissue homogenates of the Δ*sapS*-infected mice in contrast to mice that had been infected with wild-type *S. aureus* (Figure 6B), while concentrations of the pro-inflammatory cytokine TNF-α were on a comparable level in all three groups (Figure 6C). Notably, when compared to the MPO, KC, and TNF-α contents observed in kidney tissue homogenates of age-matched uninfected mice, MPO and KC levels in kidney homogenates of mice infected with a SA564 derivative harbouring a functional *sapS* were both significantly increased. MPO and KC levels in kidney tissue homogenates of Δ*sapS*-infected mice, on the other hand, did not differ statistically from the MPO and KC contents observed in kidney tissue homogenates of age-matched uninfected mice (*p* ≥ 0.1630, Kruskal–Wallis test and Dunn’s post hoc test). These data seem to indicate that *S. aureus* cells harbouring a functional *sapS*, which form abscesses in kidneys, induce a chemokine response that favours the recruitment of neutrophils to the infection site.

### 2.5. SapS Does Not Alter the Uptake Rates of S. aureus by Polymorphonuclear Neutrophils in Whole Blood

**Figure 7:**
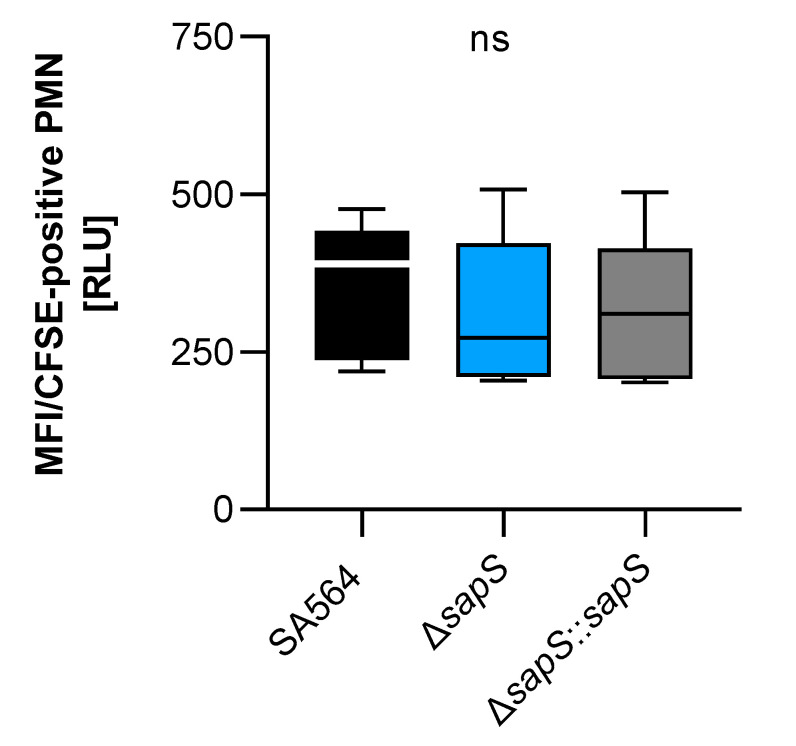
Impact of SapS on the phagocytosis of *S.aureus* strain SA564 by polymorphonuclear leukocytes (PMNs). The cells of *S. aureus* were labeled with carboxyfluorescein diacetate succinimidyl ester (CFSE) and mixed to lithium heparin-anticoagulated fresh human blood at a multiplicity of infection of 50 per PMN, and maintained at 37 °C and 1000 rpm. Using flow cytometry, as described in the Material and Methods section, the adhesion and ingestion of bacteria by PMNs was examined. The data show three biological replicates that were performed in triplicate. Data are presented as box and whisker plots showing the interquartile range (25–75%; box), median (horizontal line), and whiskers (bars; min/max). ns, not significant (Kruskal–Wallis test followed by Dunn’s post hoc test).

As we recently observed a reduced intramacrophage survival capacity of a *S. aureus* SA564 derivative lacking the protein arginine phosphatase B encoding gene *ptpB*, which was accompanied by an increased uptake rate of SA564 Δ*ptpB* mutant cells by polymorphonuclear neutrophils (PMNs) in whole blood [27], we determined whether SapS might exert a similar effect on uptake of *S. aureus* by PMNs in blood (Figure 7). However, unlike the SA564 Δ*ptpB* mutant, SA564 cells lacking *sapS* were bound/ingested to a similar extent by PMNs than SA564 isolates possessing a functional *sapS* (wild-type and cis-complemented derivative), indicating that SapS does not have a significant protective role against phagocytosis performed by PMNs in blood.

### 2.6. The Ability of S. aureus to Survive in the Presence of Oxidative Stress Is Influenced by SapS

**Figure 8:**
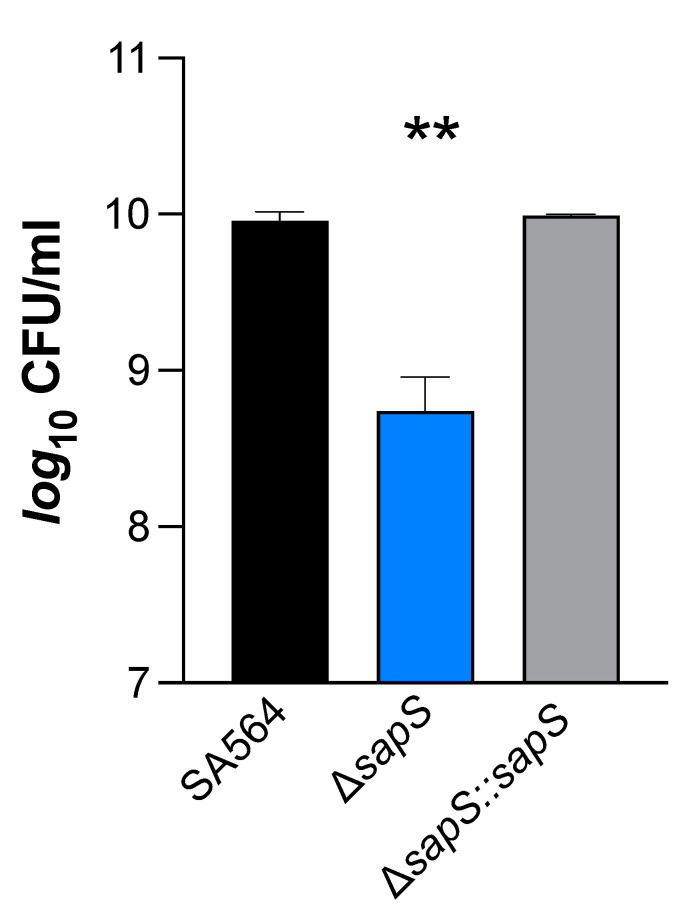
Impact of H_2_O_2_ on *S. aureus* SA564 Δ*sapS* survival. Cells of *S. aureus* SA564 (black), SA564 Δ*sapS* (blue), and SA564 Δ*sapS::sapS* (grey) were grown in TSB at 37 °C and 225 rpm for 2 h, then were challenged with 50 mM H_2_O_2_, and the CFU counts of the cultures were evaluated at 1 h after the H_2_O_2_ challenge. The data displayed are the mean + SD of four biological experiments. **, *p <* 0.01 (Ordinary One-Way ANOVA test).

*S. aureus* cells that have been phagocytosed by macrophages typically end up in phagolysosomes, where they are subjected to various stresses, including a low pH and reactive oxygen species (ROS) [35]. On the basis of our findings that *S. aureus* cells without a functional *sapS* gene had a reduced capability to survive inside RAW264.7 macrophages (Figure 1C,D), we considered the possibility that SapS might influence the capacity of *S. aureus* to respond to oxidative stress. In order to test this hypothesis, SA564 derivatives were cultivated in TSB for 2 h and then subjected to 50 mM hydrogen peroxide for an additional hour. Our results show that the ∆*sapS* derivative produced clearly lower CFU rates one hour after the H_2_O_2_ challenge than WT cells, while the *cis*-complemented strain displayed CFU rates comparable to the WT (Figure 8). These findings demonstrate that SapS has an effect on *S. aureus* capacity to cope with oxidative stress during growth, at least *in vitro*, similarly to the *S. aureus* PtpB phosphatase in this organism [27].

**Figure 9:**
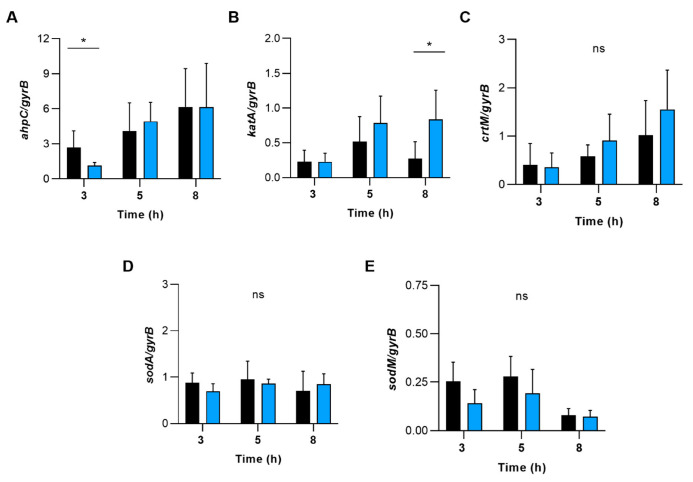
The effect of deleting the *sapS* gene in *S. aureus* on the transcription of genes that code for enzymes involved in the detoxification of reactive oxygen species (ROS). qRT-PCR was used to conduct quantitative transcript analyses of *ahpC* (**A**), *katA* (**B**), *crtM* (**C**), *sodA* (**D**) and *sodM* (**E**) in SA564 (black bars) and SA564 Δ*sapS* (blue bars) cells cultured in TSB at 37 °C and 225 rpm at the time points indicated. Quantification of the transcripts was performed in relation to the transcription of gyrase B (in copies per copy of *gyrB*). The data are shown as mean + SD of five biological replicates. ns, not significant; * *p* < 0.05 (Mann-Whitney U test between WT and mutant at a given time point).

To test whether ROS detoxifying enzymes/factors such as alkyl hydroperoxide reductase, catalase, staphyloxanthin, and superoxide dismutase might contribute to this phenotype, we determined the transcription of genes encoding for these enzymes (Figure 9). During transcription monitoring of these genes in WT and ∆s*apS* cells grown in TSB, we indeed noticed some transcriptional changes for *ahpC* and *katA* between the WT and the ∆s*apS* mutant, which, however, were all on a more minor scale (*i.e.*, ≤3-fold) and only seen for certain growth stages.

### 2.7. SapS Is Involved in S. aureus SA564 Biofilm Formation

**Figure 10:**
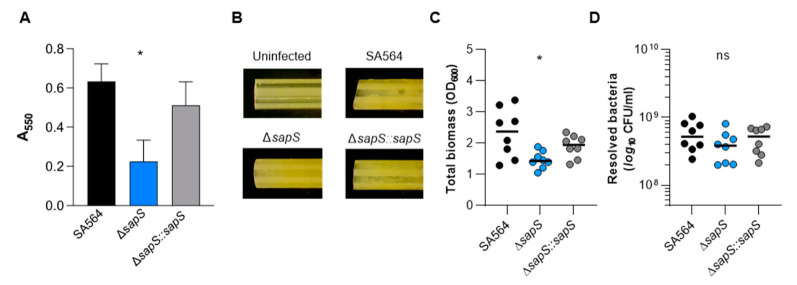
Impact of a *sapS* deletion on *S. aureus* SA564 ability to produce biofilms. (**A**) Cells of *S. aureus* SA564 (black), SA564 Δ*sapS* (blue), and SA564 Δ*sapS::sapS* (grey) were cultivated in BHI for 48 h at 37 °C in a humid atmosphere using 96-well microtiter plates. The crystal violet assay was used to evaluate the entire biofilm formation. The optical density (550 nm) and the production of biofilm are directly correlated. The presentation includes the means as well as the standard errors for a total of six different replicates. (**B**–**D**) Influence of SapS on the ability of *S. aureus* SA564 to build biofilms on medical devices. (**B**) Photographs of fragments of *S. aureus*-loaded peripheral venous catheters (PVC) taken on day 5 after the initial inoculation (6.3-fold magnification). These findings are indicative of three separate tests. An uninfected PVC fragment incubated for 5 days in sterile medium served as control. (**C**,**D**) Total biomass and CFU rates of detached biofilms were calculated by measuring the OD_600_ of the TSB solutions (**B**) and plate counting (**D**). The values of each individual experiment are represented by symbols, along with the median value (horizontal line) (n = 8). *, *p* < 0.05; ns, not significant (Kruskal–Wallis test and Dunn’s post hoc test, only the changes between SA564 and mutants are displayed).

*S. aureus* can form biofilms, which have been considered to be an important factor in its pathogenicity [36]. Therefore, we investigated whether SapS might have an impact on biofilm formation. We first tested the capacity of the strain triplet to form biofilms using a static microplate-based biofilm model as previously reported [37]. While the WT and the *cis*-complemented strain developed robust biofilms on the microtiter plate’s bottom wells after 48 h of growth in BHI, we observed a significantly lower biomass in wells inoculated with the *sapS* mutant (Figure 10A), indicating that SapS provides a favourable contribution to the capacity of *S. aureus* to produce biofilms. To confirm this effect of SapS on biofilm formation of SA564 in a more clinically relevant model, we examine the potential of the WT and its *sapS* variants to form biofilms on pieces of peripheral venous catheter (PVC) under conditions where there was an abundance of nutrients (Figure 10B–D). In this assay, all three strains generated macroscopically visible vegetation on the PVC surfaces (Figure 10B), although the vegetation formed by the WT and the *cis*-complemented derivative on the PVC surface appeared stronger than those seen with the Δ*sapS* mutant. These macroscopic observations were essentially confirmed by OD_600_ and CFU determinations (Figure 10C,D). TSB solutions harbouring the detached biofilms of the Δ*sapS* mutant produced significantly lower OD_600_ readings than biofilms of the WT (Figure 10C), and this trend was also seen for the numbers viable bacteria that were detached from the PVC tubing (Figure 10D), which were about 1/3 lower for the Δ*sapS* mutant if compared to the WT, despite the fact that this effect was not significant according to the statistics (*p* = 0.2567, Kruskal–Wallis test and Dunn’s posthoc test).

### 2.8. S. aureus SapS Phosphatase Activity Relies on a Very Specific Aspartate Residue in Its Catalytic Loop but Its Activity Is Dispensable for In Vivo Survival

**Figure 11:**
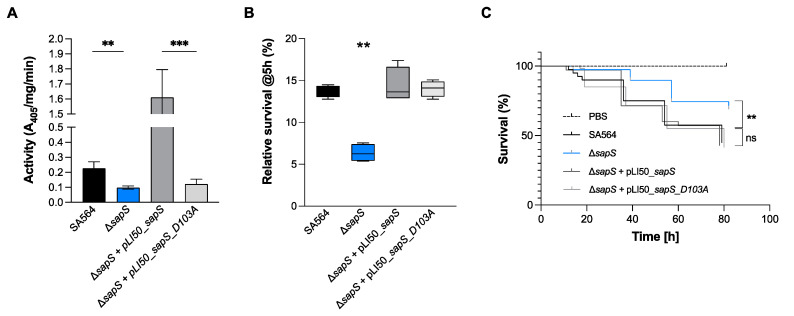
SapS phosphatase activity depends on the D103 residue and is neither implicated in the intramacrophage survival potential nor infectivity of *S. aureus* in zebrafish. (**A**) Cells of *S. aureus* SA564 (black), SA564 Δ*sapS* (blue), SA564 Δ*sapS* + pLI50_SapS-Spot (dark grey) and SA564 Δ*sapS* + pLI50_SapS_D103A-Spot (light grey) were grown in TSB at 37 °C until exponential phase, centrifuged, lysed and total crude extracts were used for phosphatase activity assays on PNPP. Data are presented as mean + SD of three biological replicates. **, *p* < 0.01; *** (Mann–Whitney U test). (**B**) Survival of *S. aureus* in infected macrophages. Survival rates are expressed as a percentage of the intracellular bacterial cell counts that were obtained immediately after the gentamicin/lysostaphin treatment, which was fixed to 100%. The data are presented as box and whisker plot showing the interquartile range (25–75%, box), the median (horizontal line) and the standard deviation (bars) of 4 different experiments. **, *p* < 0.01 (Ordinary two-way ANOVA test). (**C**) Kaplan–Meier representation of the survival of zebrafish embryos infected by injection into the duct of Cuvier with at 3 × 10^2^ CFU/nL cultured in exponential phase, or PBS (negative control) The proportion of surviving embryos (n = 50 for each, indicative of three separate experiments) is employed to express the results. **, *p* < 0.01, ns, not significant (Log-rank Mantel–Cox statistical test). hpi, hours post-infection.

SapS from *S. aureus* was previously identified as an acid phosphatase [18], and its phosphatase function was revealed by the presence of structural motifs typical for acid phosphatases (Figure S2) that are responsible for the transfer of phosphoryl groups from phosphomonoesters to water when the pH is acidic [19]. SapS belongs to the Non-Specific Acid Phosphatases (NSAPs) family, which corresponds to one subgroup of these ubiquitous phosphatases, that are secreted across the cytoplasmic membrane and exhibit optimal catalytic activity at acidic pH [38,39]. NSAPs are a separate class of phosphatases that are divided into three categories [38]. The signature of class C enzymes is composed of four aspartate residues named DDDD motif that are highly conserved (bold). These aspartate residues are located within a bipartite motif that consists of (I/V)-(V/A/L)-**D**-(I/L)-**D**-ET-(V/M)-L-X-(N/T)-X-X-Y located near the N terminus and (I/V)-(L/ M)-X-X-G-**D**-(N/T)-L-X-**D**-F close to the C terminus, both connected by a polypeptide linker region with variable length [38]. As shown in Figure S2, SapS possesses this signature motif and is therefore a Class C phosphatase. As demonstrated with orthologues [40,41,42], we assumed that the Asp residue at position 103 in SapS is mainly responsible for its phosphatase activity. To investigate the impact of Asp103 (the catalytically active Asp residue of the conserved DDDD pattern) on SapS phosphatase activity, we created the vector pLI50_*sapS*_*D103A*, harbouring a *sapS* open reading frame (orf) in which Asp103 was changed to alanine. Cells of *S. aureus* SA564, SA564 Δ*sapS*, SA564 Δ*sapS* + pLI50_*sapS* and SA564 Δ*sapS* + pLI50_*sapS_D103A* were grown in TSB at 37 °C until exponential phase, centrifuged, lysed and total crude extracts were used for phosphatase activity assays with the universal phosphate donor PNPP (Para-NitroPhenylPhosphate) (Figure 11A). In contrast to the Δ*sapS* mutant strain trans-complemented with the *sapS* wild-type gene, which produced high levels of PNPP hydrolyase activity, this was not the case with the Δ*sapS* mutant strain trans-complemented with the *sapS* gene harbouring the D103A orf, which produced a PNPP hydrolyase activity comparable to the SA564 Δ*sapS* mutant. These findings confirm earlier findings demonstrating that the *S. aureus* SapS homolog is a phosphatase [18], and strongly support our hypothesis that residue D103 is critical for the phosphatase activity of SapS.

To test whether SapS phosphatase activity is needed for *in vivo* survival of *S. aureus* within macrophages, we measured the *S. aureus* survival rates of WT and *sapS* mutant derivatives within RAW 264.7 cells at 5 h pGt (Figure 11B). Similar to our findings presented in Figure 1C,D, we again observed a significant decrease in intracellular survival of Δ*sapS* mutant cells in RAW 264.7 cells at 5 h pGt in this series of experiments, while trans-complementation of the *sapS* mutant with plasmid pLI50_*sapS* restored intracellular survival rates comparable to those of the WT strain (Figure 11B). Notably, a survival rate comparable to WT cells was also seen, when the *sapS* mutant was trans-complemented with the plasmid expressing the D103A derivative (pLI50_*sapS_D103A*), suggesting that SapS phosphatase activity is not important for this intracellular survival phenotype in macrophage cells. Given these findings, we wondered whether the phosphatase activity of SapS might play a role for infectivity of *S. aureus* in zebrafish. Similar to our observations made with the macrophage survival assays, we observed similar killing rates when larvae were infected with the WT and the trans-complemented Δ*sapS* derivative harboring plasmid pLI50_SapS_D103A (Figure 11C), indicating that SapS phosphatase activity is also not important for infectivity of *S. aureus* in this *in vivo* model of infection.

## 3. Materials and Methods

### 3.1. Bacterial Strains, Media, and Growth Conditions

Table 1 contains the bacterial strains, plasmids, and primers that were utilised for this research. The mutant strains and plasmids that were created for the purpose of this study were all sequenced to verify their authenticity. The *Escherichia coli* strains were cultured in Luria-Bertani (LB) broth at 37 °C. Isolates of *S. aureus* were either plated on Tryptic Soy Agar (TSA) or cultured in Tryptic Soy Broth (TSB) at 37 °C, 225 rpm, at a culture to flask volume of 1:10. Ampicillin was utilised at a concentration of 100 µg/mL, while chloramphenicol was employed at a concentration of 10 µg/mL for the purpose of strain construction and phenotypic selection.

### 3.2. Construction of the S. aureus sapS Deletion and Complementation Strains

Using primer pairs #34/#55 and #36/#37 (Table 2), respectively, one-kilobase segments comprising the flanking regions of the *sapS* (NWMN_0249) gene were produced by PCR from the chromosomal DNA of the *S. aureus* strain Newman. The purified PCR fragments were Gibson cloned into the EcoRI/XhoI digested pIMAY* vector, to generate pIMAY*_∆*sapS*. This fusion construct contained the first and last 15 bases of *sapS* and thus created a non-functional truncated ORF with flanking regions. The plasmid was propagated in *E. coli* IM08B [44] and electroporated into *S. aureus* strain SA564 [43]. The allelic exchange procedure was performed as described in [45] to generate the SA564 ∆*sapS* mutant strain.

In order to construct the complementation plasmid pLI50_SapS-Spot, the *sapS* gene, comprising its promoter region, was amplified by PCR with the primers #85/#77 by using chromosomal DNA of the *S. aureus* strain Newman as a template. The Spot fragment was amplified by PCR with the primers #78/86 using the pSpot2 vector as a template (Chromotek). The purified PCR fragments were Gibson cloned into the KpnI/BamHI digested pLI50 vector [47]. The pLI50_SapS_D103A-Spot plasmid derivative harbouring aspartate to alanine substitution was generated by Gibson assembly of the amplified PCR products using pLI50_SapS-Spot plasmid DNA as a template with primer pairs #85/#116 and #117/#86, respectively. The pLI50_SapS-Spot derivative plasmids were transformed in *E. coli* IM08B and electroporated into *S. aureus* strain SA564 ∆*sapS*.

For the construction of the integrative complementation plasmid pJB38NWMN2930_SapS-Spot, the *sapS* gene, including its promoter region, and the fused Spot-tag at the C-terminus, was amplified using primers #85/#86 and pLI50_SapS-Spot plasmid DNA as a template. The purified PCR fragment was phosphorylated and cloned into the EcoRV digested pJB38NWMN2930 vector [46]. The allelic exchange protocol was realised as previously detailed to generate the SA564 ∆*sapS::sapS* complemented strain [46].

### 3.3. Macrophage Culture and Infection

The mouse macrophage cell line RAW 264.7 (mouse leukemic monocyte macro-phage, ATCC TIB-71) was grown in Dulbecco’s modified Eagle’s medium (DMEM) (Thermo Fisher Scientific, Dreieich, Germany), which was complemented with 10% foetal calf serum (Thermo Fisher Scientific), at 37 °C in a humid atmosphere with 5% carbon dioxide. For infected macrophages, *S. aureus* strain SA564 and its derivatives were grown in TSB medium until they reached the mid-exponential growth stage (OD600 = 0.7–0.9). After being collected and centrifuged for 5 min at 4000 rpm, the bacteria were then washed in sterile PBS, centrifuged for 4 min at 10,000 rpm, and lastly reconstituted in sterile PBS. RAW 264.7 cells were infected with *S. aureus* SA564 or its derivatives at a multiplicity of infection (MOI) of 20:1 (bacteria/cells) and then incubated for one hour at 37 °C and 5% carbon dioxide. There were 2.5 × 10^5^ cells in each well of the 24-well plates. Afterwards, cells were rinsed one more time with PBS, and then the extracellular bacteria were eradicated by incubating the cells for 30 min with gentamicin at a concentration of 100 µg/mL. After being treated with gentamicin, macrophages were washed two times in PBS (T0), followed by an incubation period of 5h in fresh medium containing 5 µg/mL of lysostaphin. After this incubation period, macrophages were lysed with 0.1% Triton X-100 in PBS. Intracellular bacteria was determined by plating out serially diluted macrophage lysates on TSB agar plates, which were incubated for 24 h at 37 °C. For macrophage lysates immunoprecipitation, at 3 h or 5 h pGt. Infected macrophages were lysed in 0.1 %Triton X-100, and centrifuged at 14,000× *g*. At 3 h and 5 h pGt, infected macrophages were lysed in 0.1% Triton X-100 and centrifuged at 14,000× *g* for macrophage lysates immunoprecipitation. Anti-Spot magnetic beads (Chromotek) were used to immunoprecipitate the resulting supernatants, and the bacterial pellets corresponding to intracellular bacteria were resuspended in an equivalent volume of lysis buffer.

### 3.4. Infection of Zebrafish Embryos

The *S. aureus* SA564 and derivative strains were cultivated overnight at 37 °C in TSB medium. Cultures were diluted 1:20 and grown in TSB medium until they reached the mid-exponential growth phase (OD600 = 0.7–0.9). Once the bacteria had been centrifuged for 10 min at 4000 rpm, they were resuspended in PBS at the desired concentration (around 2 × 10^8^ bacteria/mL). The injected suspension was prepared by addition of phenol Red (Sigma, P0290-100 mL, 0.5% phenol red solution diluted 0,1 % in the injected solution) and fluorescent dye (Dextran, tetramethylrhodamine, 10,000 MW, anionic, D-1868 Invitrogen, 30 µM in the injected solution) to help following the injection procedure. The GAB zebrafish line was used in the experiments, which were performed in fish water at 28 °C. Embryos were infected by injecting 1 nL of bacterial suspensions into the duct of Cuvier of 50 hpf embryos, which had been previously dechorionated and sedated with tricaine (0.3 mg/mL). Each injected embryo was then placed in a separate well of 96 wells microtiter plate. In order to verify the quantity of bacteria present in each injection, the same amount was injected into 200 µL of PBS, and the number of CFU was quantified using TSB agar plates. The number of dead embryos was visually counted in order to evaluate survival kinetics following infection depending on the disappearance of heartbeat.

### 3.5. Murine Abscess Model

Experiments on animals were conducted with the authorization of the local State Review Board in the state of Saarland, Germany, and were conducted in accordance with the national and European standards for the ethical and decent animal experimentation. Both the production of the bacterial inoculum and the infection of the mice were performed as previously described [27]. Briefly, 100 µL of bacterial culture that contained ~107 colony forming units (CFU) of exponential growth phase cells were injected retro-orbitally into female C57BL/6N mice (Charles River, Sulzfeld, Germany) between the ages of 8 and 10 weeks old while they were anesthetised with 3.5% isoflurane (Baxter, Unterschleißheim, Germany). The infected mice were treated with a dose of carprofen (5 mg/kg; Zoetis, Berlin, Germany) shortly after infection. At day four after infection, mice were killed, and their livers and kidneys were collected. The organs were weight normalised and homogenised in PBS (Thermo Fisher, Dreieich, Germany). Afterwards, successive dilutions of the homogenates were plated on blood agar plates in order to enumerate the CFU rates in the organs. Tissue homogenates were subsequently centrifuged for 5 min at 5000× *g* and 4 °C, and supernatants used for MPO, KC, and TNF-α determination utilising commercial ELISA kits from R&D System (Wiesbaden, Germany), according to the manufacturer’s instructions, and as described in [48]. Tissue homogenates obtained from 10- to 12-week-old C57BL/6N mice served as uninfected controls.

### 3.6. Determination of Immune Cell Contents in Blood of Mice

Blood was collected when mice were sacrificed at day 4 post infection. Four 10- to 12-week-old C57BL/6N mice served as uninfected control. Red blood cells (RBCs) were lysed with ACK RBCs lysis buffer (Thermo Fisher Scientific, Karlsruhe, Germany). The remaining blood cells were fixed with 1% paraformaldehyde in PBS solution and resuspended in FACS buffer composed of 0.1 mM EDTA and 1% fetal bovine serum (FBS) dissolved in PBS. Blood cells were then incubated in Fc-blocking solution (CD16/CD32 Antibody, Thermo Fisher Scientific) for 40 min at 4 °C, and subsequently stained for 45 min at 4 °C with the following fluorochrome-conjugated antibodies: F4/80-PECy7 (clone BM8), CD45-PerCP-Cy5.5 (clone 30-F11), CD11b-FITC (clone M1/70), CD86-PE (clone GL1), CD11c-Super Bright 436 (clone N418) LY6G-APC (1A8-Ly6g), CD3- eFluor™ 450 (clone 17A2), CD4-FITC (clone RM4-5), CD8-PECy7 (clone 53-6.7), CD45R-PE-eFluor™ 610 (clone RA3-6B2), and NK1.1- APC (clone PK136). All fluorochrome-conjugated antibodies were brought from eBioscience (Thermo Fisher Scientific). Flow cytometric analysis was performed using the BD FACSverse machine, and the data collected were analysed with the software Flowjo v10.6.2 (Becton Dickinson, Franklin Lakes, NJ, USA).

### 3.7. Analysis of Phagocytosis in Human Whole Blood

The ingestion of *S. aureus* cells by PMNs in whole blood was performed in accordance with the procedures outlined in [49]. Overnight cultures of *S. aureus* strains were inoculated into fresh TSB and grown at 37 °C and 225 rpm to mid-exponential growth-phase. Bacterial cells were harvested by centrifugation at 10,000× *g* for 5 min and washed three times with PBS. Bacterial cells were next stained with a 50 µM carboxy fluorescein diacetate succinimidyl ester (CFSE; Invitrogen, Darmstadt, Germany); PBS solution for 15 min at 37 °C and 1000 rpm. After that, the CFSE-stained bacterial cells were washed once more with PBS three times to remove any remaining unbound dye, and the OD600 was adjusted to a value of 1. The PMN content of the blood sample was assessed by using the RAL DIFF-QUICK kit (RAL Diagnostics, Martillac, France) in accordance with the recommendations provided by the manufacturer after fresh human whole blood was drawn from healthy donors and anticoagulated with lithium heparin (S-Monovette; Sarstedt, Nümbrecht, Germany). Blood samples were inoculated with fluorescently labelled bacteria at a MOI of 50 per PMN, and cultivated in the dark at 37 °C and 1000 rpm for 30 min. The cell suspensions were transferred into 5 mL round bottom polystyrene tubes (BD), the erythrocytes were lysed by the addition of FACS lysis solution (BD), and the lysed cell debris were eliminated by centrifugation at 450× *g* for 5 min. Flow cytometry was performed on the cell pellets after they were resuspended in PBS that included 2% foetal calf serum (PAA, Pasching, Germany) and 0.05% sodium azide. The analysis was performed on a FACSCalibur (BD) device. The CellQuest Pro Software version 4.02 (BD) was used to gate PMNs, and the mean fluorescence intensity (MFI) of each PMN was recorded. This value represents the amount of bacteria that were either adhered to or ingested by the leukocyte.

### 3.8. Measurement of Gene Expression by qRT-PCR

Following the procedures described previously [50], RNA isolation, cDNA synthesis, and qRT-PCR were performed using the primer pairs provided in Table 2. Quantification of the transcripts was performed in relation to the transcription of gyrase B (in copies per copy of *gyrB*).

### 3.9. Cell Fractionation and Immunoblotting

Bacteria were cultivated up to the exponential phase in TSB at 37 °C with shaking (160 rpm) and cultures were centrifuged after 2 h of growth at 4000 rpm for 10 min. The culture supernatant was transferred to another tube and Spot-fusion proteins were immunoprecipitated with anti-Spot beads (Chromotek) at 4 °C on a rotating wheel for 3 h (IP). The bacterial pellet was washed twice with phosphate buffered saline (PBS) and resuspended in digestion buffer (50 mM Tris-HCl, 20 mM MgCl_2_, 30% [wt/vol] raffinose; pH 7.5) containing complete mini-EDTA-free protease inhibitors (Roche). Cell wall proteins were digested with lysostaphin (200 µg/mL) for 30 min at 37 °C to solubilise them. Centrifugation at 5000× *g* for 15 min was used to collect the protoplasts, and the supernatant was preserved as the cell wall fraction (W). Protoplast pellets were rinsed in digestion buffer once, sedimented (5000× *g*, 15 min), and then resuspended in ice-cold lysis buffer (50 mM Tris-HCl [pH 7.5]) with protease inhibitors and DNase (80 µg/mL). Freeze-thaw cycles were used to lyse the protoplasts. Membranes were separated from cell lysates by ultracentrifugation at 150,000× *g* for 40 min. While the pellet (membrane fraction, M) was suspended in Tris-buffer, the supernatant (cytosolic fraction, C) was transferred to a new tube. Protein samples were migrated on an SDS-PAGE gel and then transferred to polyvinylidene fluoride (PVDF) membranes for immunoblotting. In TBST (25 mM Tris/HCl pH 7.6, 150 mM NaCl, 0.05% TWEEN), the membrane was blocked with 50% human serum (Sigma, ref. H3667) and 3% BSA for an overnight incubation at 4 °C. The membrane was then incubated for 1 h with monoclonal mouse anti-Spot antibody (1:5000, Chromotek) diluted in 3% BSA in TBST. Afterwards, the membrane was washed 3 times in TBST and the secondary HRP coupled donkey anti-mouse antibody (1:5000, Jackson Immuno Research ref. 715-035-151) was incubated for 1 h in 3% BSA in TBST. After washing the membrane three times in TBST, the HRP signal was detected by Pierce ECL Western Blotting substrate.

### 3.10. SapS Phosphatase Activity Assay

On crude extracts of *S. aureus* SA564 and its derivatives, the phosphatase activity was measured using a technique based on the detection of p-nitrophenol (PNP), which is produced by the cleavage of p-nitrophenyl phosphate (PNPP). Tests were conducted in a buffer at pH 5.0 containing 4 mM PNPP, and 100 mM sodium citrate. 35 µg of crude extract was added to start the reaction, which was then allowed to sit in 96-well microplates for 25 min. Using a Tecan fluorimeter (Tecan, Model Spark, Grodig, Austria GmbH ) microplate reader, the phosphatase reaction was observed at 405 nm (absorption maximum of the produced PNP). All experiments were done at least in triplicate.

### 3.11. H_2_O_2_ Susceptibility Assays

After culturing bacterial cells in TSB for 2 h at 37 °C and 225 rpm, the bacteria were exposed to H_2_O_2_ to a final concentration of 50 mM for 1 h. Plate counting was used to estimate the CFU rates of the cultures after the allotted period of incubation had passed.

### 3.12. Biofilm Assays

Crystal Violet Biofilm Assay: Biofilm formation was assessed using the method outlined in [37]. After an overnight incubation at 37 °C and a dilution to obtain a final optical density of 0.1 at OD_600_, biofilm development was assessed by incubating 200 µL of bacterial cultures in 96-well microtiter plates in BHI. The plates were placed in a humid container and incubated at 37 °C for a period of 48 h. Following incubation, adherent cells were washed with PBS, allowed to air dry, and then resuspended with a crystal violet (0.1%) solution and left at room temperature for 20 min. In order to dissolve the biofilm, 100 µL of acetic acid at a concentration of 33% was applied to each well, and the measurement of absorbance was performed using a Tecan equipment (Tecan, Model Spark, Grodig, Austria GmbH) at OD_550_ nm.

Following the methodology given in [51], the formation of biofilms on medical devices was conducted under dynamic conditions. PVC (Venflon Pro Safety 18 G; Becton Dickinson, USA) fragments measuring 1 cm in length were positioned into reaction tubes containing 1 mL of TSB. These tubes were then inoculated with 5 × 10^5^ CFU of TSB-washed bacterial cells obtained from the exponential growth phase (2.5 h of growth at 37 °C and 225 rpm). An uninfected PVC fragment served as control. PVC pieces were kept in an incubator for 5 days at 37 °C and 150 rpm, and the medium was changed out every 24 h. On day five, PVC fragments were inserted into fresh reaction tubes that were filled with 1 mL of TSB. Biofilms were removed from the surface of the catheter and resolved by sonication (50 watts for 5 min) followed by vortexing for 1 min. Plate counting was used to assess CFU rates and OD_600_ readings were used to determine the biomass of dissolved biofilms.

### 3.13. Statistical Analyses

Prism 6.01 software (GraphPad, USA) was utilised to determine the statistical significance of differences among groups, as shown in the figure legends.

## 4. Conclusions

*S. aureus* is able to produce a broad variety of disease patterns because it has a vast arsenal of virulence factors [52,53], which provide the pathogen with the ability to penetrate or escape the immune system, attach to surfaces, and create detrimental toxic effects on the host. Interestingly, our recent research showed that PtpA, a low-molecular-weight protein tyrosine phosphatase, that is produced by *S. aureus* was secreted during growth and is related to the pathogenicity of this pathogen [16]. Our findings, which are presented here, show that the knockout of *sapS* in the *S. aureus* clinical isolate SA564 affects the survival *in vitro* and virulence in infection animal models. This leads us to conclude that the secreted SapS phosphatase is significant for the process of infection as well as the intracellular survival of *S. aureus*. Furthermore, mutational studies show that the phosphatase activity of SapS is not needed for the infectivity promoting activity of this protein, leaving the question open as to how Saps contributes on the molecular level favourably to *S. aureus* pathogenicity. However, given its clear impact on biofilm formation and infectivity of *S. aureus*, SapS represents a novel and promising new target for therapeutic development to fight against this well-known human pathogen.

## Figures and Tables

**Table 1:** Strains, plasmids, and primers used in this study.

Strain	Description	Reference or Source
***S. aureus***		
SA564	*S. aureus* clinical isolate, wild type	[43]
SA564 Δ*sapS*	SA564 deletion mutant of the *sapS* gene	This study
SA564 Δ*sapS::sapS*	SA564 Δ*sapS* complemented with the pJB38-NWMN2930_SapS-Spot integrative plasmid	This study
SA564 Δ*sapS* + pLI50_SapS-Spot	SA564 Δ*sapS* complemented with the pLI50_SapS-Spot plasmid	This study
SA564 Δ*sapS* + pLI50_SapS_D103A-Spot	SA564 Δ*sapS* complemented with the pLI50_SapS_D103A-Spot	This study
***E. coli***		
TOP10	*E. coli* derivative ultra-competent cells used for general cloning	Invitrogen
IM08B	*E. coli* DC10B derivative harbouring *hsdS* of *S. aureus* strain NRS384, Δ*dcm*	[44]
**Plasmids**		
pIMAY	*E. coli–S. aureus* temperature-sensitive suicide shuttle vector, *pheS* counterselection; *cat*	[45]
pIMAY_Δ*sapS*	pIMAY derivative harbouring the genomic regions flanking *sapS*; *cat*	This study
pJB38-NWMN2930	*E. coli–S. aureus* temperature-sensitive shuttle vector for chromosomal integration containing the Newman genetic region between genes 29 and 30; *bla, cat*	[46]
pJB38NWMN2930_SapS-Spot	pJB38-NWMN2930 derivative used to integrate and express C-terminal Spot-tagged fusion of *S. aureus* SapS; *bla, cat*	This study
pLI50	*E. coli*-*S. aureus* shuttle vector for native expression; *bla, cat*	[47]
pLI50_*sapS*	pLI50 derivative used to express C-terminal Spot-tagged fusion of *S. aureus* SapS; *bla, cat*	This study
pLI50_*sapS_D103A*	pLI50 derivative used to express C-terminal Spot-tagged fusion of *S. aureus* SapS_D103A; *bla, cat*	This study

**Table 2:** Primers used in this study.

Primers	5′ to 3′ Sequence
#34	CGGGCTGCAGGAATTTAAACTAATCCAGTAAACGA
#55	TTTAACTTCGCCTGTTGAAATTTTATTCATCTTATCACCTCATG
#36	ATGAATAAAATTTCAACAGGCGAAGTTAAATAATA
#37	CGGGCCCCCCCTCGATGTAGCTGAAATGACAAATA
#85	ATTCGAGCTCGGTACCGTAAATAAGAGATAGCACA
#77	TGGCCCTGATGACCCTTTAACTTCGCCTGTTTTAG
#78	ACAGGCGAAGTTAAAGGGTCATCAGGGCCAGATCG
#86	CGACTCTAGAGGATCCTAAGAACTCCAATGTGATA
#116	TTCATCTAAAGCCAAAGCAATAGCTAACTTATG
#117	CATAAGTTAGCTATTGCTTTGGCTTTAGATGAA
**qRT PCR primers**	
*ahpC* for	TCCAACTGAATTAGAAGACT
*ahpC* rev	GAGAATACATTTACGCCTAAT
*crtM* for	ACAGTAGGTGAAGTATTGAC
*crtM* rev	ATCGTATGTCTGATGTGTTT
*gyrA* for	GACTGATGCCGATGTGGA
*gyrA* rev	AACGGTGGCTGTGCAATA
*katA* for	AATGGACAATGTATATTCAAGT
*katA* rev	ATCAAATGGATTATCTTTATGGT
*sapS* for	ATAATTCTCCATATCAAGGCTAT
*sapS* rev	TGGGAAAGGTTTATTATGTATTG
*sodA* for	ACCAAGATAATCCATTAACTGA
*sodA* rev	ATTTTAGGTAATAAGCGTGTTC
*sodM* for	CCAAGATAATCCATTAACAGAA
*sodM* rev	CCAAACATCAAATAGTAAGATTG

## Data Availability

The datasets generated during and/or analysed during the current study are available from the corresponding author on reasonable request.

## Supplementary Materials

The following supporting information can be downloaded at https://www.mdpi.com/article/10.3390/ijms232214031/s1.
