# Supplementary material for: Characterization of the Secreted Acid Phosphatase SapS Reveals a Novel Virulence Factor of Staphylococcus aureus That Contributes to Survival and Virulence in Mice
Source: Int J Mol Sci. 2022 Nov 14;23(22):14031. doi: 10.3390/ijms232214031 (PMC9692844; doi:10.3390/ijms232214031)
Supplement: Supplementary file 1 [file ijms-23-14031-s001.zip › ijms-1991256-supplementary.pdf]

Supplementary Figure S1

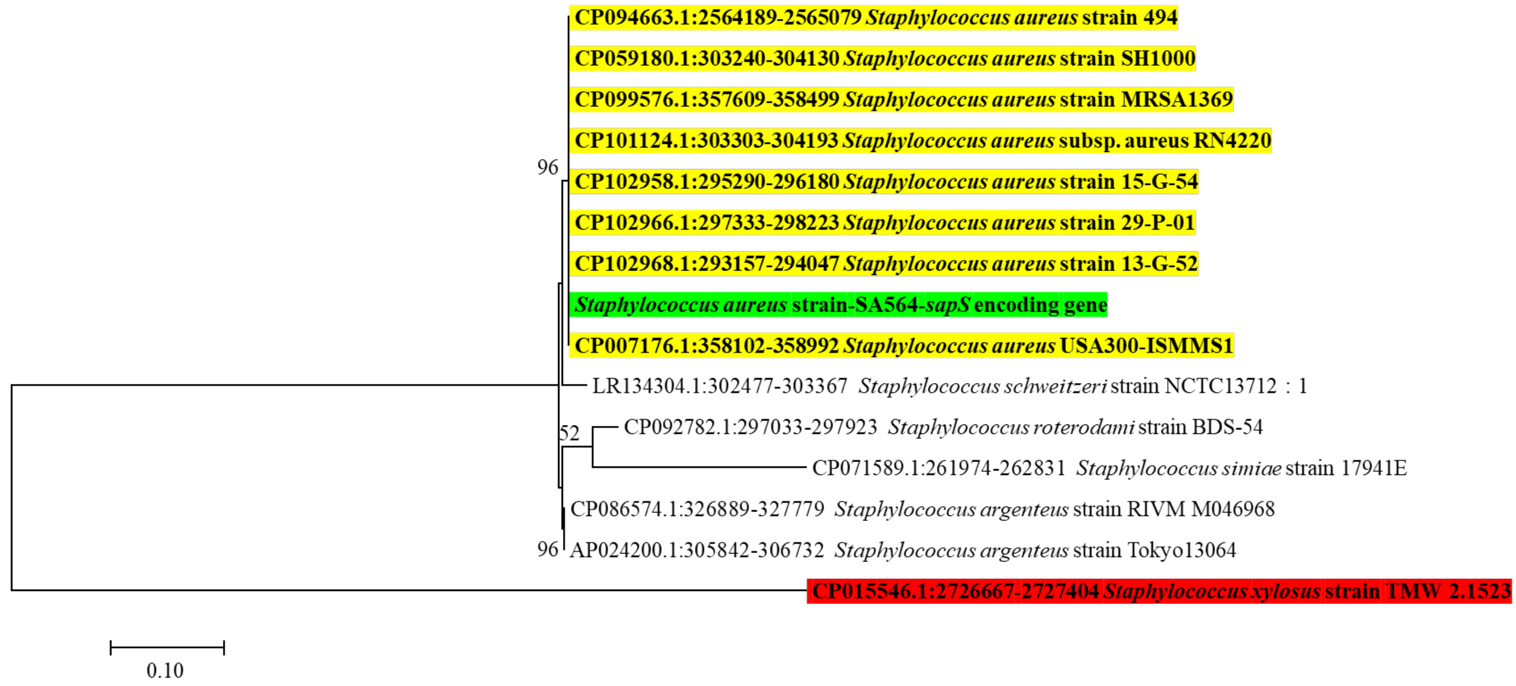

Supplementary Figure S1: Phylogenetic analysis-based on complete sequence of *Staphylococcus aureus* strain SA564 *sapS* gene with the eight hit-blast and other most similar sequences from other *Staphylococcus* species including *Staphylococcus xylosus* as an out of the groupe. Eight *S. aureus* sequences recovered from the NBCI GenBank nucleotide sequence database (http://www.ncbi.nlm.nih.gov/nucleotide/), indicated by a bold font and yellow filling and the identified *sapS* gene in green filling were incorporated in the phylogeny reconstruction in addition to other *Staphylococcus* species. The evolutionary history was inferred by using the Maximum Likelihood method based on the Kimura 2-parameter model. The initial tree(s) for the heuristic search were obtained automatically by applying Neighbor-Join and BioNJ algorithms to a matrix of pairwise distances estimated using the Maximum Composite Likelihood (MCL) approach, and then selecting the topology with superior log likelihood value. The evolutionary analyses were conducted in MEGA7 (version 7.0.26), including 701 positions in the final dataset from 15 nucleotide sequences and all positions containing gaps and missing data were eliminated. The tree is drawn to scale, with branch lengths measured in the number of substitutions per site. Bootstrap values >50% are labeled on the tree.

Supplementary Figure S2

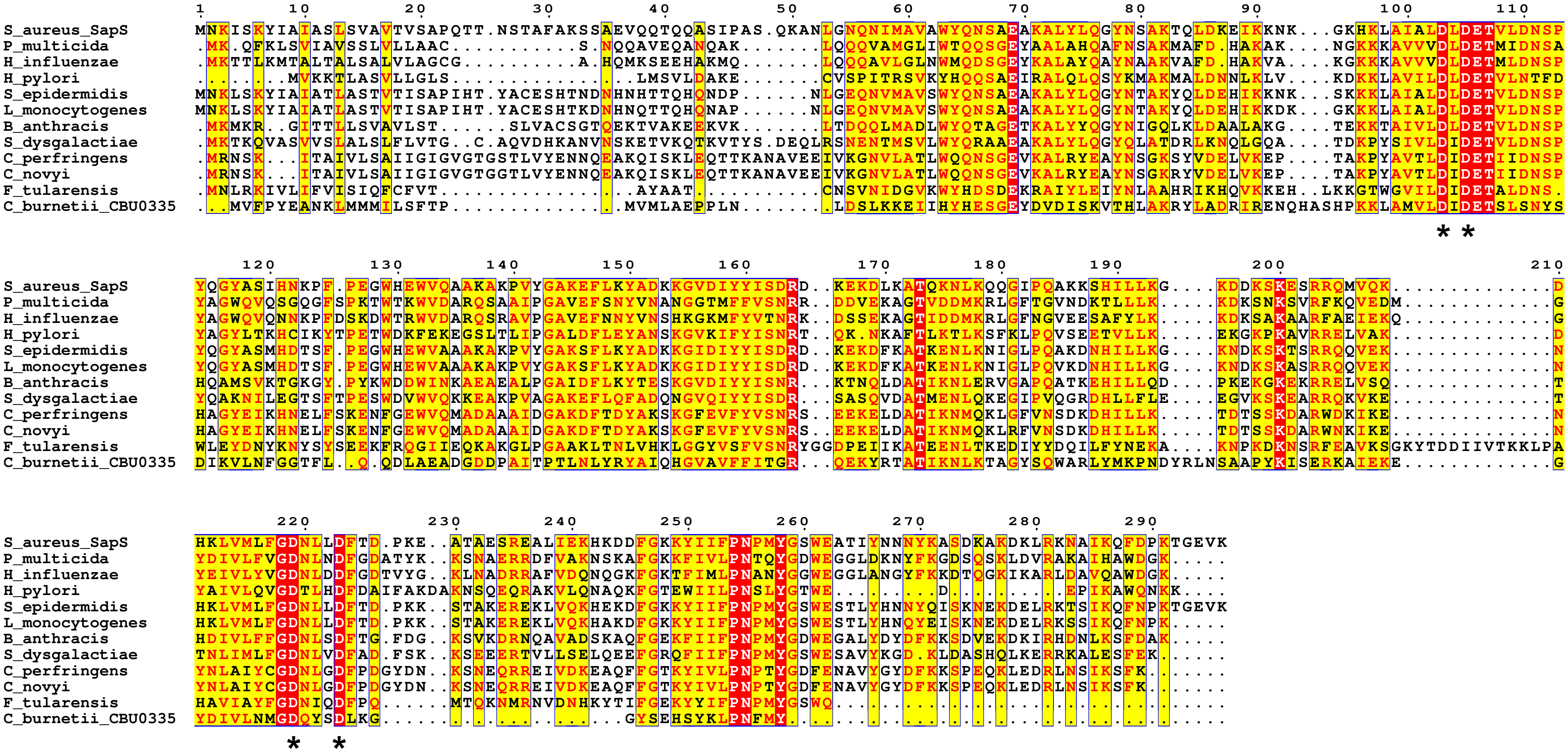

Supplementary Figure S2: Multiple sequence alignment of acid phosphatase proteins from bacterial origin. The top amino acid sequence of the alignment is that of the *S. aureus* SapS protein from this study. Directly above the *S. aureus* sequence are black numbers (from 1 to 290 in multiples of 10) designating the residue number. Other sequences are from *Pasteurella multocida, Haemophilus influenzae, Helicobacter pylori, Staphylococcus epidermidis, Listeria monocytogenes, Bacillus anthracis, Streptococcus dysgalactiae, Clostridium perfringens, Clostridium novyi, Francisella tularensis* and *Coxiella burnetii*. Residues identically conserved in all sequences are denoted by white letters on a red background. Other regions of high sequence conservation are indicated by boxed red letters on a yellow background. Black stars under the alignment indicate residues of the DDDD motif. This figure was created with ESPript (Robert & Gouet, 2014).
